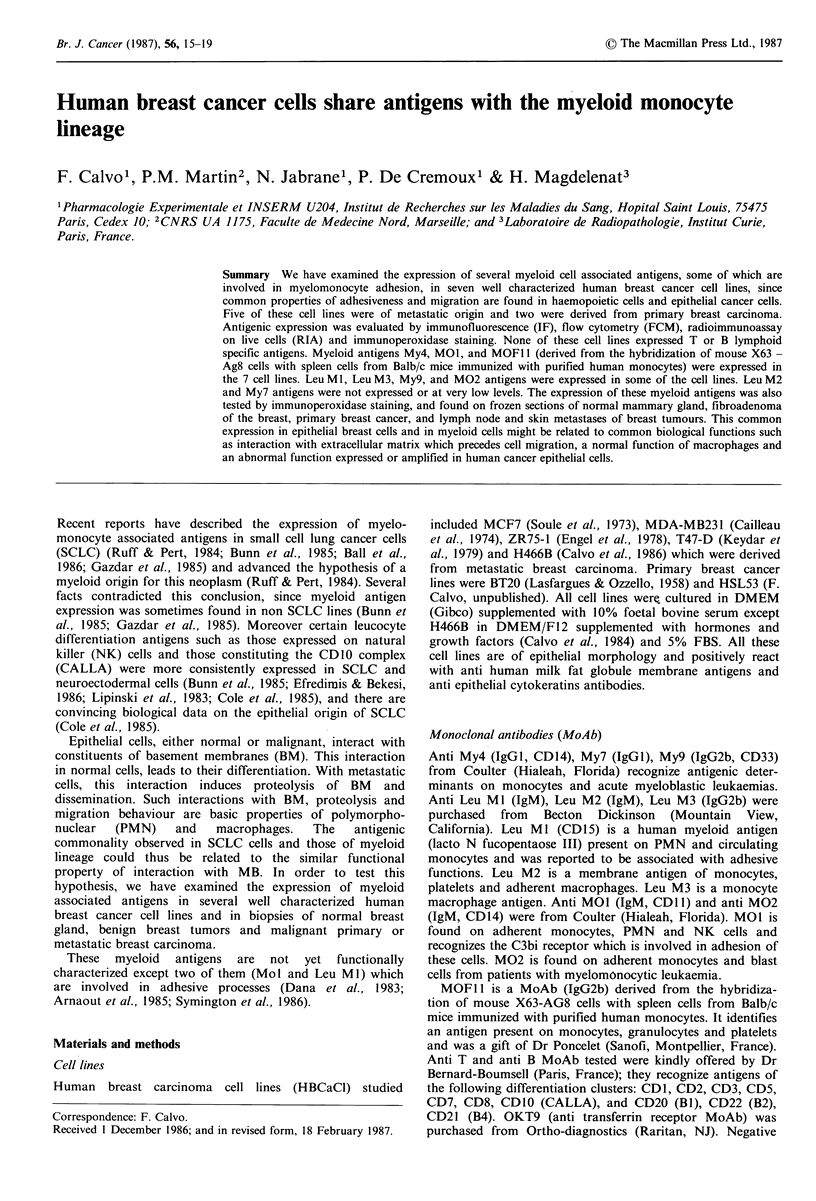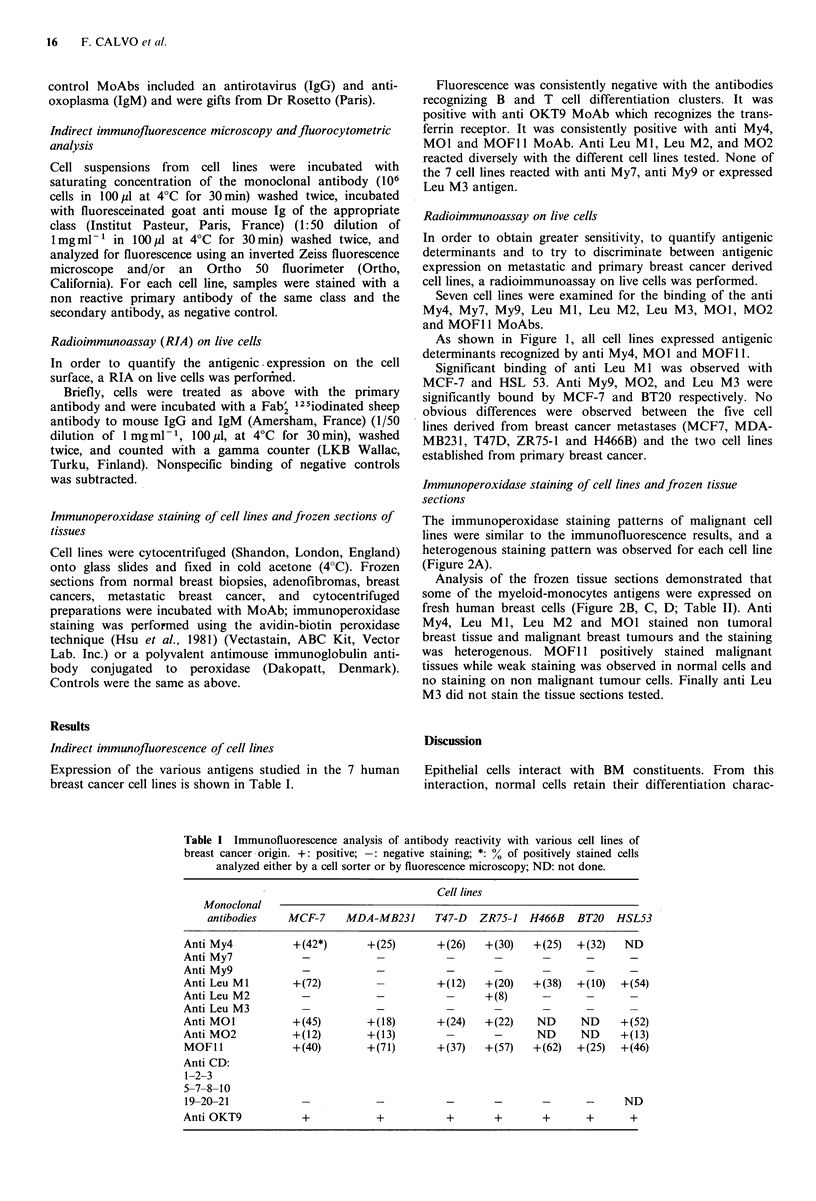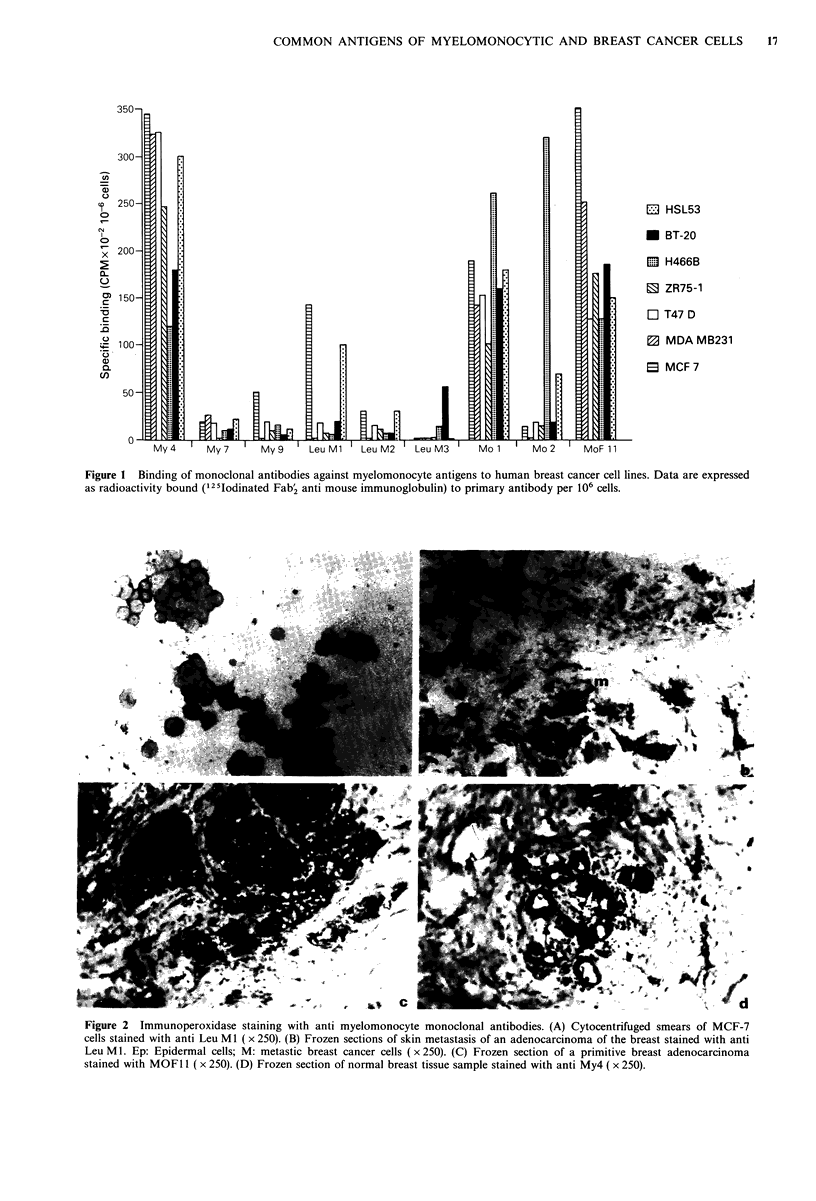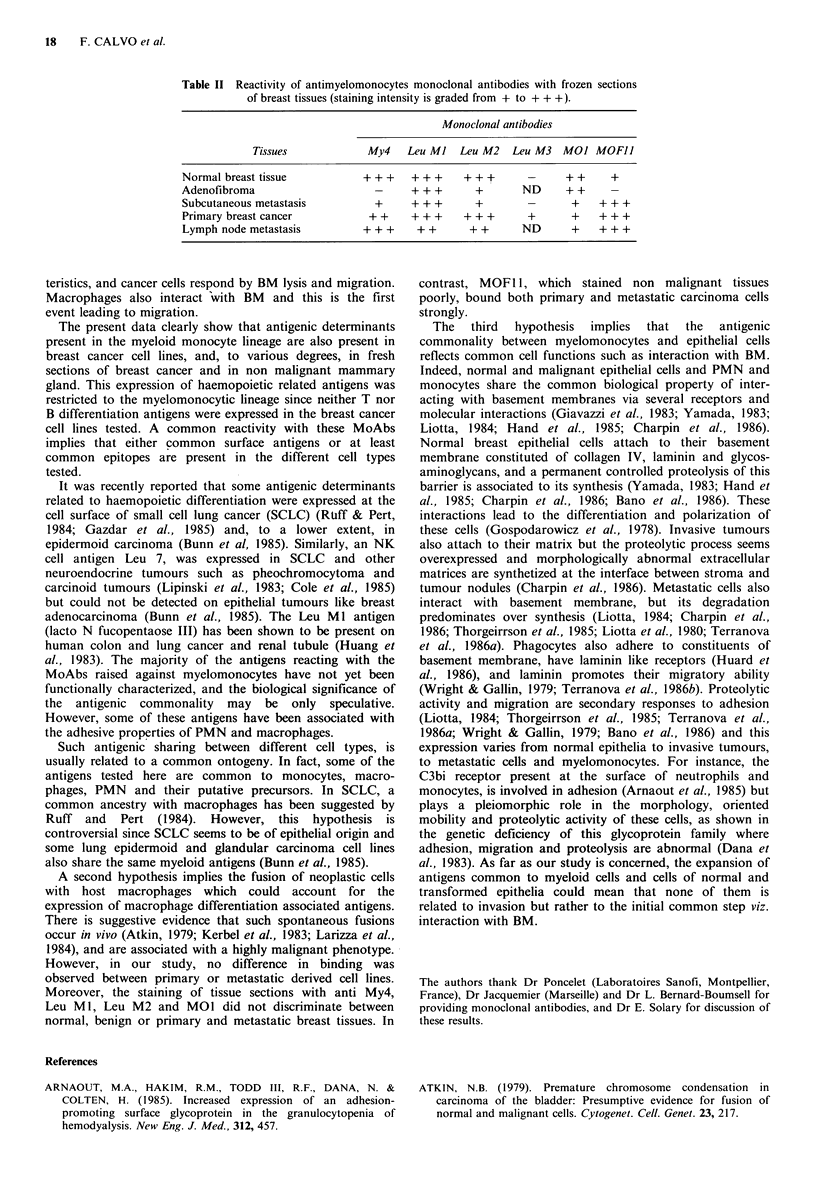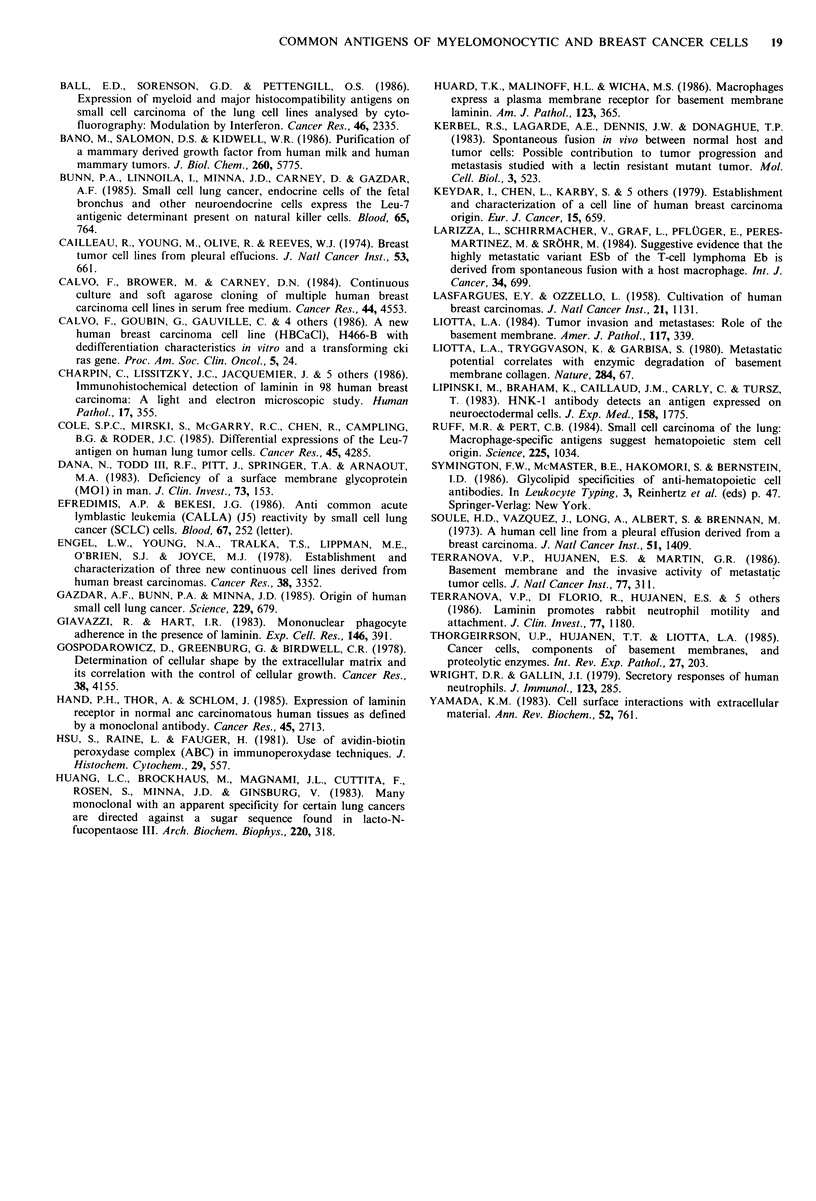# Human breast cancer cells share antigens with the myeloid monocyte lineage.

**DOI:** 10.1038/bjc.1987.145

**Published:** 1987-07

**Authors:** F. Calvo, P. M. Martin, N. Jabrane, P. De Cremoux, H. Magdelenat

## Abstract

**Images:**


Br. J. Cancer (1987), 56, 15–19 © The Macmillan Press Ltd., 1987

### Human breast cancer cells share antigens with the myeloid monocyte lineage

F. Calvo[1], P.M. Martin[2], N. Jabrane[1], P. De Cremoux[1] & H. Magdelenat[3]

[1]*Pharmacologie Experimentale et INSERM U204, Institut de Recherches sur les Maladies du Sang, Hopital Saint Louis, 75475 Paris, Cedex 10;* [2]*CNRS UA 1175, Faculte de Medecine Nord, Marseille; and* [3]*Laboratoire de Radiopathologie, Institut Curie, Paris, France.*

**Summary** We have examined the expression of several myeloid cell associated antigens, some of which are involved in myelomonocyte adhesion, in seven well characterized human breast cancer cell lines, since common properties of adhesiveness and migration are found in haemopoietic cells and epithelial cancer cells. Five of these cell lines were of metastatic origin and two were derived from primary breast carcinoma. Antigenic expression was evaluated by immunofluorescence (IF), flow cytometry (FCM), radioimmunoassay on live cells (RIA) and immunoperoxidase staining. None of these cell lines expressed T or B lymphoid specific antigens. Myeloid antigens My4, MO1, and MOF11 (derived from the hybridization of mouse X63 – Ag8 cells with spleen cells from Balb/c mice immunized with purified human monocytes) were expressed in the 7 cell lines. Leu M1, Leu M3, My9, and MO2 antigens were expressed in some of the cell lines. Leu M2 and My7 antigens were not expressed or at very low levels. The expression of these myeloid antigens was also tested by immunoperoxidase staining, and found on frozen sections of normal mammary gland, fibroadenoma of the breast, primary breast cancer, and lymph node and skin metastases of breast tumours. This common expression in epithelial breast cells and in myeloid cells might be related to common biological functions such as interaction with extracellular matrix which precedes cell migration, a normal function of macrophages and an abnormal function expressed or amplified in human cancer epithelial cells.

Recent reports have described the expression of myelomonocyte associated antigens in small cell lung cancer cells (SCLC) (Ruff & Pert, 1984; Bunn *et al.*, 1985; Ball *et al.*, 1986; Gazdar *et al.*, 1985) and advanced the hypothesis of a myeloid origin for this neoplasm (Ruff & Pert, 1984). Several facts contradicted this conclusion, since myeloid antigen expression was sometimes found in non SCLC lines (Bunn *et al.*, 1985; Gazdar *et al.*, 1985). Moreover certain leucocyte differentiation antigens such as those expressed on natural killer (NK) cells and those constituting the CD10 complex (CALLA) were more consistently expressed in SCLC and neuroectodermal cells (Bunn *et al.*, 1985; Efredimis & Bekesi, 1986; Lipinski *et al.*, 1983; Cole *et al.*, 1985), and there are convincing biological data on the epithelial origin of SCLC (Cole *et al.*, 1985).

Epithelial cells, either normal or malignant, interact with constituents of basement membranes (BM). This interaction in normal cells, leads to their differentiation. With metastatic cells, this interaction induces proteolysis of BM and dissemination. Such interactions with BM, proteolysis and migration behaviour are basic properties of polymorphonuclear (PMN) and macrophages. The antigenic commonality observed in SCLC cells and those of myeloid lineage could thus be related to the similar functional property of interaction with MB. In order to test this hypothesis, we have examined the expression of myeloid associated antigens in several well characterized human breast cancer cell lines and in biopsies of normal breast gland, benign breast tumors and malignant primary or metastatic breast carcinoma.

These myeloid antigens are not yet functionally characterized except two of them (Mol and Leu M1) which are involved in adhesive processes (Dana *et al.*, 1983; Arnaout *et al.*, 1985; Symington *et al.*, 1986).

### Materials and methods

### *Cell lines*

Human breast carcinoma cell lines (HBCaCl) studied included MCF7 (Soule *et al.*, 1973), MDA-MB231 (Cailleau *et al.*, 1974), ZR75-1 (Engel *et al.*, 1978), T47-D (Keydar *et al.*, 1979) and H466B (Calvo *et al.*, 1986) which were derived from metastatic breast carcinoma. Primary breast cancer lines were BT20 (Lasfargues & Ozzello, 1958) and HSL53 (F. Calvo, unpublished). All cell lines were cultured in DMEM (Gibco) supplemented with 10% foetal bovine serum except H466B in DMEM/F12 supplemented with hormones and growth factors (Calvo *et al.*, 1984) and 5% FBS. All these cell lines are of epithelial morphology and positively react with anti human milk fat globule membrane antigens and anti epithelial cytokeratins antibodies.

### *Monoclonal antibodies (MoAb)*

Anti My4 (IgG1, CD14), My7 (IgG1), My9 (IgG2b, CD33) from Coulter (Hialeah, Florida) recognize antigenic determinants on monocytes and acute myeloblastic leukaemias. Anti Leu M1 (IgM), Leu M2 (IgM), Leu M3 (IgG2b) were purchased from Becton Dickinson (Mountain View, California). Leu M1 (CD15) is a human myeloid antigen (lacto N fucopentaose III) present on PMN and circulating monocytes and was reported to be associated with adhesive functions. Leu M2 is a membrane antigen of monocytes, platelets and adherent macrophages. Leu M3 is a monocyte macrophage antigen. Anti MO1 (IgM, CD11) and anti MO2 (IgM, CD14) were from Coulter (Hialeah, Florida). MO1 is found on adherent monocytes, PMN and NK cells and recognizes the C3bi receptor which is involved in adhesion of these cells. MO2 is found on adherent monocytes and blast cells from patients with myelomonocytic leukaemia.

MOF11 is a MoAb (IgG2b) derived from the hybridization of mouse X63-AG8 cells with spleen cells from Balb/c mice immunized with purified human monocytes. It identifies an antigen present on monocytes, granulocytes and platelets and was a gift of Dr Poncelet (Sanofi, Montpellier, France). Anti T and anti B MoAb tested were kindly offered by Dr Bernard-Boumsell (Paris, France); they recognize antigens of the following differentiation clusters: CD1, CD2, CD3, CD5, CD7, CD8, CD10 (CALLA), and CD20 (B1), CD22 (B2), CD21 (B4). OKT9 (anti transferrin receptor MoAb) was purchased from Ortho-diagnostics (Raritan, NJ). Negative

Correspondence: F. Calvo.
Received 1 December 1986; and in revised form, 18 February 1987.

control MoAbs included an antirotavirus (IgG) and anti-oxoplasma (IgM) and were gifts from Dr Rosetto (Paris).

*Indirect immunofluorescence microscopy and fluorocytometric analysis*

Cell suspensions from cell lines were incubated with saturating concentration of the monoclonal antibody ($10^6$ cells in 100 $\mu$l at 4°C for 30 min) washed twice, incubated with fluoresceinated goat anti mouse Ig of the appropriate class (Institut Pasteur, Paris, France) (1:50 dilution of 1 mg $ml^{-1}$ in 100 $\mu$l at 4°C for 30 min) washed twice, and analyzed for fluorescence using an inverted Zeiss fluorescence microscope and/or an Ortho 50 fluorimeter (Ortho, California). For each cell line, samples were stained with a non reactive primary antibody of the same class and the secondary antibody, as negative control.

*Radioimmunoassay (RIA) on live cells*

In order to quantify the antigenic expression on the cell surface, a RIA on live cells was performed.

Briefly, cells were treated as above with the primary antibody and were incubated with a $Fab'_2$ $^{125}$iodinated sheep antibody to mouse IgG and IgM (Amersham, France) (1/50 dilution of 1 mg $ml^{-1}$, 100 $\mu$l, at 4°C for 30 min), washed twice, and counted with a gamma counter (LKB Wallac, Turku, Finland). Nonspecific binding of negative controls was subtracted.

*Immunoperoxidase staining of cell lines and frozen sections of tissues*

Cell lines were cytocentrifuged (Shandon, London, England) onto glass slides and fixed in cold acetone (4°C). Frozen sections from normal breast biopsies, adenofibromas, breast cancers, metastatic breast cancer, and cytocentrifuged preparations were incubated with MoAb; immunoperoxidase staining was performed using the avidin-biotin peroxidase technique (Hsu *et al.*, 1981) (Vectastain, ABC Kit, Vector Lab. Inc.) or a polyvalent antimouse immunoglobulin antibody conjugated to peroxidase (Dakopatt, Denmark). Controls were the same as above.

### Results

*Indirect immunofluorescence of cell lines*

Expression of the various antigens studied in the 7 human breast cancer cell lines is shown in Table I.

Fluorescence was consistently negative with the antibodies recognizing B and T cell differentiation clusters. It was positive with anti OKT9 MoAb which recognizes the transferrin receptor. It was consistently positive with anti My4, MO1 and MOF11 MoAb. Anti Leu M1, Leu M2, and MO2 reacted diversely with the different cell lines tested. None of the 7 cell lines reacted with anti My7, anti My9 or expressed Leu M3 antigen.

*Radioimmunoassay on live cells*

In order to obtain greater sensitivity, to quantify antigenic determinants and to try to discriminate between antigenic expression on metastatic and primary breast cancer derived cell lines, a radioimmunoassay on live cells was performed.

Seven cell lines were examined for the binding of the anti My4, My7, My9, Leu M1, Leu M2, Leu M3, MO1, MO2 and MOF11 MoAbs.

As shown in Figure 1, all cell lines expressed antigenic determinants recognized by anti My4, MO1 and MOF11.

Significant binding of anti Leu M1 was observed with MCF-7 and HSL 53. Anti My9, MO2, and Leu M3 were significantly bound by MCF-7 and BT20 respectively. No obvious differences were observed between the five cell lines derived from breast cancer metastases (MCF7, MDA-MB231, T47D, ZR75-1 and H466B) and the two cell lines established from primary breast cancer.

*Immunoperoxidase staining of cell lines and frozen tissue sections*

The immunoperoxidase staining patterns of malignant cell lines were similar to the immunofluorescence results, and a heterogenous staining pattern was observed for each cell line (Figure 2A).

Analysis of the frozen tissue sections demonstrated that some of the myeloid-monocytes antigens were expressed on fresh human breast cells (Figure 2B, C, D; Table II). Anti My4, Leu M1, Leu M2 and MO1 stained non tumoral breast tissue and malignant breast tumours and the staining was heterogenous. MOF11 positively stained malignant tissues while weak staining was observed in normal cells and no staining on non malignant tumour cells. Finally anti Leu M3 did not stain the tissue sections tested.

### Discussion

Epithelial cells interact with BM constituents. From this interaction, normal cells retain their differentiation charac-

**Table I** Immunofluorescence analysis of antibody reactivity with various cell lines of breast cancer origin. +: positive; −: negative staining; *: % of positively stained cells analyzed either by a cell sorter or by fluorescence microscopy; ND: not done.

| Monoclonal antibodies | Cell lines | | | | | | |
|---|---|---|---|---|---|---|---|
| | *MCF-7* | *MDA-MB231* | *T47-D* | *ZR75-1* | *H466B* | *BT20* | *HSL53* |
| Anti My4 | +(42*) | +(25) | +(26) | +(30) | +(25) | +(32) | ND |
| Anti My7 | − | − | − | − | − | − | − |
| Anti My9 | − | − | − | − | − | − | − |
| Anti Leu M1 | +(72) | − | +(12) | +(20) | +(38) | +(10) | +(54) |
| Anti Leu M2 | − | − | − | +(8) | − | − | − |
| Anti Leu M3 | − | − | − | − | − | − | − |
| Anti MO1 | +(45) | +(18) | +(24) | +(22) | ND | ND | +(52) |
| Anti MO2 | +(12) | +(13) | − | − | ND | ND | +(13) |
| MOF11 | +(40) | +(71) | +(37) | +(57) | +(62) | +(25) | +(46) |
| Anti CD: | | | | | | | |
| 1–2–3 | | | | | | | |
| 5–7–8–10 | | | | | | | |
| 19–20–21 | − | − | − | − | − | − | ND |
| Anti OKT9 | + | + | + | + | + | + | + |

**Figure 1** Binding of monoclonal antibodies against myelomonocyte antigens to human breast cancer cell lines. Data are expressed as radioactivity bound ([125]Iodinated Fab'$_2$ anti mouse immunoglobulin) to primary antibody per $10^6$ cells.

**Figure 2** Immunoperoxidase staining with anti myelomonocyte monoclonal antibodies. (A) Cytocentrifuged smears of MCF-7 cells stained with anti Leu M1 ( × 250). (B) Frozen sections of skin metastasis of an adenocarcinoma of the breast stained with anti Leu M1. Ep: Epidermal cells; M: metastic breast cancer cells ( × 250). (C) Frozen section of a primitive breast adenocarcinoma stained with MOF11 ( × 250). (D) Frozen section of normal breast tissue sample stained with anti My4 ( × 250).

**Table II** Reactivity of antimyelomonocytes monoclonal antibodies with frozen sections of breast tissues (staining intensity is graded from + to + + +).

| Tissues | Monoclonal antibodies | | | | | |
|---|---|---|---|---|---|---|
| | *My4* | *Leu M1* | *Leu M2* | *Leu M3* | *MO1* | *MOF11* |
| Normal breast tissue | + + + | + + + | + + + | − | + + | + |
| Adenofibroma | − | + + + | + | ND | + + | − |
| Subcutaneous metastasis | + | + + + | + | − | + | + + + |
| Primary breast cancer | + + | + + + | + + + | + | + | + + + |
| Lymph node metastasis | + + + | + + | + + | ND | + | + + + |

teristics, and cancer cells respond by BM lysis and migration. Macrophages also interact with BM and this is the first event leading to migration.

The present data clearly show that antigenic determinants present in the myeloid monocyte lineage are also present in breast cancer cell lines, and, to various degrees, in fresh sections of breast cancer and in non malignant mammary gland. This expression of haemopoietic related antigens was restricted to the myelomonocytic lineage since neither T nor B differentiation antigens were expressed in the breast cancer cell lines tested. A common reactivity with these MoAbs implies that either common surface antigens or at least common epitopes are present in the different cell types tested.

It was recently reported that some antigenic determinants related to haemopoietic differentiation were expressed at the cell surface of small cell lung cancer (SCLC) (Rüff & Pert, 1984; Gazdar *et al.*, 1985) and, to a lower extent, in epidermoid carcinoma (Bunn *et al*, 1985). Similarly, an NK cell antigen Leu 7, was expressed in SCLC and other neuroendocrine tumours such as pheochromocytoma and carcinoid tumours (Lipinski *et al.*, 1983; Cole *et al.*, 1985) but could not be detected on epithelial tumours like breast adenocarcinoma (Bunn *et al.*, 1985). The Leu M1 antigen (lacto N fucopentaose III) has been shown to be present on human colon and lung cancer and renal tubule (Huang *et al.*, 1983). The majority of the antigens reacting with the MoAbs raised against myelomonocytes have not yet been functionally characterized, and the biological significance of the antigenic commonality may be only speculative. However, some of these antigens have been associated with the adhesive properties of PMN and macrophages.

Such antigenic sharing between different cell types, is usually related to a common ontogeny. In fact, some of the antigens tested here are common to monocytes, macrophages, PMN and their putative precursors. In SCLC, a common ancestry with macrophages has been suggested by Ruff and Pert (1984). However, this hypothesis is controversial since SCLC seems to be of epithelial origin and some lung epidermoid and glandular carcinoma cell lines also share the same myeloid antigens (Bunn *et al.*, 1985).

A second hypothesis implies the fusion of neoplastic cells with host macrophages which could account for the expression of macrophage differentiation associated antigens. There is suggestive evidence that such spontaneous fusions occur *in vivo* (Atkin, 1979; Kerbel *et al.*, 1983; Larizza *et al.*, 1984), and are associated with a highly malignant phenotype. However, in our study, no difference in binding was observed between primary or metastatic derived cell lines. Moreover, the staining of tissue sections with anti My4, Leu M1, Leu M2 and MO1 did not discriminate between normal, benign or primary and metastatic breast tissues. In contrast, MOF11, which stained non malignant tissues poorly, bound both primary and metastatic carcinoma cells strongly.

The third hypothesis implies that the antigenic commonality between myelomonocytes and epithelial cells reflects common cell functions such as interaction with BM. Indeed, normal and malignant epithelial cells and PMN and monocytes share the common biological property of interacting with basement membranes via several receptors and molecular interactions (Giavazzi *et al.*, 1983; Yamada, 1983; Liotta, 1984; Hand *et al.*, 1985; Charpin *et al.*, 1986). Normal breast epithelial cells attach to their basement membrane constituted of collagen IV, laminin and glycosaminoglycans, and a permanent controlled proteolysis of this barrier is associated to its synthesis (Yamada, 1983; Hand *et al.*, 1985; Charpin *et al.*, 1986; Bano *et al.*, 1986). These interactions lead to the differentiation and polarization of these cells (Gospodarowicz *et al.*, 1978). Invasive tumours also attach to their matrix but the proteolytic process seems overexpressed and morphologically abnormal extracellular matrices are synthetized at the interface between stroma and tumour nodules (Charpin *et al.*, 1986). Metastatic cells also interact with basement membrane, but its degradation predominates over synthesis (Liotta, 1984; Charpin *et al.*, 1986; Thorgeirrson *et al.*, 1985; Liotta *et al.*, 1980; Terranova *et al.*, 1986*a*). Phagocytes also adhere to constituents of basement membrane, have laminin like receptors (Huard *et al.*, 1986), and laminin promotes their migratory ability (Wright & Gallin, 1979; Terranova *et al.*, 1986*b*). Proteolytic activity and migration are secondary responses to adhesion (Liotta, 1984; Thorgeirrson *et al.*, 1985; Terranova *et al.*, 1986*a*; Wright & Gallin, 1979; Bano *et al.*, 1986) and this expression varies from normal epithelia to invasive tumours, to metastatic cells and myelomonocytes. For instance, the C3bi receptor present at the surface of neutrophils and monocytes, is involved in adhesion (Arnaout *et al.*, 1985) but plays a pleiomorphic role in the morphology, oriented mobility and proteolytic activity of these cells, as shown in the genetic deficiency of this glycoprotein family where adhesion, migration and proteolysis are abnormal (Dana *et al.*, 1983). As far as our study is concerned, the expansion of antigens common to myeloid cells and cells of normal and transformed epithelia could mean that none of them is related to invasion but rather to the initial common step *viz.* interaction with BM.

The authors thank Dr Poncelet (Laboratoires Sanofi, Montpellier, France), Dr Jacquemier (Marseille) and Dr L. Bernard-Boumsell for providing monoclonal antibodies, and Dr E. Solary for discussion of these results.

**References**

ARNAOUT, M.A., HAKIM, R.M., TODD III, R.F., DANA, N. & COLTEN, H. (1985). Increased expression of an adhesion-promoting surface glycoprotein in the granulocytopenia of hemodyalysis. *New Eng. J. Med.*, **312**, 457.

ATKIN, N.B. (1979). Premature chromosome condensation in carcinoma of the bladder: Presumptive evidence for fusion of normal and malignant cells. *Cytogenet. Cell. Genet.* **23**, 217.

BALL, E.D., SORENSON, G.D. & PETTENGILL, O.S. (1986). Expression of myeloid and major histocompatibility antigens on small cell carcinoma of the lung cell lines analysed by cytofluorography: Modulation by Interferon. *Cancer Res.*, **46**, 2335.

BANO, M., SALOMON, D.S. & KIDWELL, W.R. (1986). Purification of a mammary derived growth factor from human milk and human mammary tumors. *J. Biol. Chem.*, **260**, 5775.

BUNN, P.A., LINNOILA, I., MINNA, J.D., CARNEY, D. & GAZDAR, A.F. (1985). Small cell lung cancer, endocrine cells of the fetal bronchus and other neuroendocrine cells express the Leu-7 antigenic determinant present on natural killer cells. *Blood*, **65**, 764.

CAILLEAU, R., YOUNG, M., OLIVE, R. & REEVES, W.J. (1974). Breast tumor cell lines from pleural effucions. *J. Natl Cancer Inst.*, **53**, 661.

CALVO, F., BROWER, M. & CARNEY, D.N. (1984). Continuous culture and soft agarose cloning of multiple human breast carcinoma cell lines in serum free medium. *Cancer Res.*, **44**, 4553.

CALVO, F., GOUBIN, G., GAUVILLE, C. & 4 others (1986). A new human breast carcinoma cell line (HBCaCl), H466-B with dedifferentiation characteristics *in vitro* and a transforming cki ras gene. *Proc. Am. Soc. Clin. Oncol.*, **5**, 24.

CHARPIN, C., LISSITZKY, J.C., JACQUEMIER, J. & 5 others (1986). Immunohistochemical detection of laminin in 98 human breast carcinoma: A light and electron microscopic study. *Human Pathol.*, **17**, 355.

COLE, S.P.C., MIRSKI, S., McGARRY, R.C., CHEN, R., CAMPLING, B.G. & RODER, J.C. (1985). Differential expressions of the Leu-7 antigen on human lung tumor cells. *Cancer Res.*, **45**, 4285.

DANA, N., TODD III, R.F., PITT, J., SPRINGER, T.A. & ARNAOUT, M.A. (1983). Deficiency of a surface membrane glycoprotein (MO1) in man. *J. Clin. Invest.*, **73**, 153.

EFREDIMIS, A.P. & BEKESI, J.G. (1986). Anti common acute lymblastic leukemia (CALLA) (J5) reactivity by small cell lung cancer (SCLC) cells. *Blood*, **67**, 252 (letter).

ENGEL, L.W., YOUNG, N.A., TRALKA, T.S., LIPPMAN, M.E., O'BRIEN, S.J. & JOYCE, M.J. (1978). Establishment and characterization of three new continuous cell lines derived from human breast carcinomas. *Cancer Res.*, **38**, 3352.

GAZDAR, A.F., BUNN, P.A. & MINNA, J.D. (1985). Origin of human small cell lung cancer. *Science*, **229**, 679.

GIAVAZZI, R. & HART, I.R. (1983). Mononuclear phagocyte adherence in the presence of laminin. *Exp. Cell. Res.*, **146**, 391.

GOSPODAROWICZ, D., GREENBURG, G. & BIRDWELL, C.R. (1978). Determination of cellular shape by the extracellular matrix and its correlation with the control of cellular growth. *Cancer Res.*, **38**, 4155.

HAND, P.H., THOR, A. & SCHLOM, J. (1985). Expression of laminin receptor in normal anc carcinomatous human tissues as defined by a monoclonal antibody. *Cancer Res.*, **45**, 2713.

HSU, S., RAINE, L. & FAUGER, H. (1981). Use of avidin-biotin peroxydase complex (ABC) in immunoperoxydase techniques. *J. Histochem. Cytochem.*, **29**, 557.

HUANG, L.C., BROCKHAUS, M., MAGNAMI, J.L., CUTTITA, F., ROSEN, S., MINNA, J.D. & GINSBURG, V. (1983). Many monoclonal with an apparent specificity for certain lung cancers are directed against a sugar sequence found in lacto-N-fucopentaose III. *Arch. Biochem. Biophys.*, **220**, 318.

HUARD, T.K., MALINOFF, H.L. & WICHA, M.S. (1986). Macrophages express a plasma membrane receptor for basement membrane laminin. *Am. J. Pathol.*, **123**, 365.

KERBEL, R.S., LAGARDE, A.E., DENNIS, J.W. & DONAGHUE, T.P. (1983). Spontaneous fusion *in vivo* between normal host and tumor cells: Possible contribution to tumor progression and metastasis studied with a lectin resistant mutant tumor. *Mol. Cell. Biol.*, **3**, 523.

KEYDAR, I., CHEN, L., KARBY, S. & 5 others (1979). Establishment and characterization of a cell line of human breast carcinoma origin. *Eur. J. Cancer*, **15**, 659.

LARIZZA, L., SCHIRRMACHER, V., GRAF, L., PFLÜGER, E., PERES-MARTINEZ, M. & SRÖHR, M. (1984). Suggestive evidence that the highly metastatic variant ESb of the T-cell lymphoma Eb is derived from spontaneous fusion with a host macrophage. *Int. J. Cancer*, **34**, 699.

LASFARGUES, E.Y. & OZZELLO, L. (1958). Cultivation of human breast carcinomas. *J. Natl Cancer Inst.*, **21**, 1131.

LIOTTA, L.A. (1984). Tumor invasion and metastases: Role of the basement membrane. *Amer. J. Pathol.*, **117**, 339.

LIOTTA, L.A., TRYGGVASON, K. & GARBISA, S. (1980). Metastatic potential correlates with enzymic degradation of basement membrane collagen. *Nature*, **284**, 67.

LIPINSKI, M., BRAHAM, K., CAILLAUD, J.M., CARLY, C. & TURSZ, T. (1983). HNK-1 antibody detects an antigen expressed on neuroectodermal cells. *J. Exp. Med.*, **158**, 1775.

RUFF, M.R. & PERT, C.B. (1984). Small cell carcinoma of the lung: Macrophage-specific antigens suggest hematopoietic stem cell origin. *Science*, **225**, 1034.

SYMINGTON, F.W., McMASTER, B.E., HAKOMORI, S. & BERNSTEIN, I.D. (1986). Glycolipid specificities of anti-hematopoietic cell antibodies. In *Leukocyte Typing*, **3**, Reinhertz *et al.* (eds) p. 47. Springer-Verlag: New York.

SOULE, H.D., VAZQUEZ, J., LONG, A., ALBERT, S. & BRENNAN, M. (1973). A human cell line from a pleural effusion derived from a breast carcinoma. *J. Natl Cancer Inst.*, **51**, 1409.

TERRANOVA, V.P., HUJANEN, E.S. & MARTIN, G.R. (1986). Basement membrane and the invasive activity of metastatic tumor cells. *J. Natl Cancer Inst.*, **77**, 311.

TERRANOVA, V.P., DI FLORIO, R., HUJANEN, E.S. & 5 others (1986). Laminin promotes rabbit neutrophil motility and attachment. *J. Clin. Invest.*, **77**, 1180.

THORGEIRRSON, U.P., HUJANEN, T.T. & LIOTTA, L.A. (1985). Cancer cells, components of basement membranes, and proteolytic enzymes. *Int. Rev. Exp. Pathol.*, **27**, 203.

WRIGHT, D.R. & GALLIN, J.I. (1979). Secretory responses of human neutrophils. *J. Immunol.*, **123**, 285.

YAMADA, K.M. (1983). Cell surface interactions with extracellular material. *Ann. Rev. Biochem.*, **52**, 761.